# Plutonium signatures in refractory fallout support a Chernobyl nuclear jet hypothesis

**DOI:** 10.1007/s10967-025-10541-0

**Published:** 2025-11-20

**Authors:** Malcolm. J. Joyce, Colin Boxall, Marcus Christl, Patrick Collins-Price, Pawel Gaca, Philip Gautschi, Francis Livens, Argaia Madina, Kirk T. Semple, Phillip Warwick, Richard Wilbraham

**Affiliations:** 1 School of Engineering, Lancaster University, Lancaster, LA1 4YW UK; 2 Laboratory of Ion Beam Physics, ETH Zürich, HPK, G23, Otto-Stern-Weg 5, CH-8093 Zurich, Switzerland; 3 National Oceanography Centre, University of Southampton, Southampton, European Way, Southampton, SO14 3ZH UK; 4 Department of Earth & Environmental Sciences, The University of Manchester, Oxford Road, Manchester, M13 9PL UK; 5 Lancaster Environment Centre, Lancaster University, Lancaster, LA1 4YQ UK

**Keywords:** Chernobyl, Environmental plutonium, Refractory fallout, Accelerator mass spectrometry

## Abstract

Measurements of refractory and volatile components in soils from Northern England confirm ^244^Pu/^239^Pu consistent with the global average but refractory ^240^Pu/^239^Pu significantly higher but consistent with Chernobyl fallout, i.e., 0.390 ± 0.006. Refractory formation suggests temperatures > 3000°C, consistent with atmospheric injection by nuclear-driven explosions rather than  by steam or hydrogen. Volatile ^240^Pu/^239^Pu is consistent with the global average, 0.181 ± 0.002, and hence lower-temperature formation in the chemical explosions and subsequent fire. This hypothesis is supported by ^239^Pu fission and capture cross-section enhancement due to the S-wave resonance at ~ 0.3 eV. A further calibration with a ^240^Pu standard is recommended to substantiate these observations.

**Supplementary Information:**

The online version contains supplementary material available at 10.1007/s10967-025-10541-0.

## Introduction

Thermal-neutron mediated nuclear explosion(s) are thought to have contributed to the destruction of the Chernobyl reactor, alongside exothermic chemical (steam/hydrogen) reactions and prior to the fire that followed, because the scale of the destruction exceeds what would be expected with the energy released by chemical reactions alone [1]. The possibility of an explosion is supported further by the observation of characteristic ^133^Xe and ^133m^Xe activity ratios detected in the air very soon after the accident, some 1000 km distant in Cherepovets [2, 3], that match the yield of a rapid nuclear excursion rather than that of steady-state power production in a thermal spectrum reactor.

The distance at which these ratios were detected also suggests an injection of fallout material or ‘jet’ [4] at a considerably higher altitude than that of the bulk reactor debris, consistent with the faster evolution and greater magnitude of a nuclear event compared to the chemically driven issue that followed. This is corroborated by consistent seismic measurements and observations of a blue flash above the plant before the subsequent fire [4]. It has been postulated that the latter may have been due the cooling of debris from high temperatures, i.e., ~ 7000 K, ejected high into the atmosphere by such a nuclear event [4]. However, independent evidence for such temperatures has not been reported.

Understanding this is important, as the energy and duration of a release, i.e., its power, influences the altitude attained and hence the range over which fine, i.e., sub-micron, particulate debris might have been dispersed and deposited, as per observations at nuclear weapons test sites [5]. This research reports evidence of an isotopic plutonium signature in trace residues consistent with Chernobyl fallout, found in a refractory component sourced a significant distance from the plant, consistent with a high-altitude injection and the high-temperature formation hypothesis.

## Theory

In addition to the short-lived xenon activities referred to above, isotopic plutonium ratios can serve as forensic indicators, i.e., of the neutron flux and spectrum, of the environment responsible for producing the associated isotopic material. These are usually quantified as the ratio of a given isotopic abundance to that of a majority isotope, where for plutonium the latter is usually ^239^Pu [6].

Observation of the full range of plutonium isotopes is feasible in fallout from a high-flux nuclear explosion involving plutonium. Those isotopes heavier than ^239^Pu are formed via successive neutron capture reactions, through to ^244^Pu. Plutonium-244 is extraordinarily rare in the environment, as per a typical global average atom ratio [7] of ^244^Pu/^239^Pu = (14.4 ± 1.5) × 10^–5^, i.e., 1 in ~ 7000 plutonium atoms. Nuclear weapons fallout is characterised by ^244^Pu/^239^Pu levels of typically an order of magnitude higher than the global average, at > 10^–4^. Conversely, whilst ^240^Pu accounts for just under 1 in 5 atoms globally in soil, cf., accepted global averages of ^240^Pu/^239^Pu = 0.176 ± 0.014 [8], and 0.180 ± 0.007 [9] (errors expressed at 1σ), ^240^Pu arisings in weapons fallout are typically low relative to the global average, cf., Montebello, ^240^Pu/^239^Pu ~ 0.04 [10].

Thermal spectrum nuclear reactors, such as Unit 4 at Chernobyl, do not produce ^244^Pu because their neutron flux is too low to overcome the short-lived decay $t_{1/2}$(= 4.96 h) of the intermediary isotope, ^243^Pu, relative to that in nuclear weapons tests. However, the production of ^240^Pu is not so impeded and it can be produced in relatively significant quantities, dependent on burn-up and the operational history of the reactor in question; some cases in point include: ^240^Pu/^239^Pu ~ 0.23 for Magnox [11], 0.39–0.56 for Chernobyl [12–15, 17–19] and 0.27–0.48 for reactor fallout associated with Fukushima [20]. At the other extreme, fast breeder reactors might typically exhibit characteristically low ^240^Pu levels, cf., ^240^Pu/^239^Pu of 0.03–0.05 for Dounreay [6, 21]. This is because the ^239^Pu(n,γ)^240^Pu cross-section at fast neutron energies is lower (by a factor of ~ 5000) than for thermal neutrons. This undermines ^240^Pu production compared to that for thermal neutrons, as in the production of weapons grade material, cf., Mayak [22].

Fallout can comprise both refractory and volatile components, with the former arising when radioactive species of interest have a boiling point higher than the solidification temperature of material constituting bulk debris, where the latter might serve as a carrier material by which radioactivity might be dispersed. This causes the radioactivity to be entrained throughout the carrier substance. In contrast, volatile species arise when the radioactive residues have a lower boiling point causing them to adhere instead to the outside of solid particles of debris [23]. The possibility of there being a heterogenous distribution comprising both refractory and volatile components specifically in Chernobyl fallout, stemming from the initial explosion and subsequent fire, respectively, has been reported [24].

An important distinction of refractory and volatile phases is that a significantly more rigorous preparatory dissolution procedure is necessary to penetrate refractory particulate volumes than for the volatile phase. Otherwise, much of the radioactivity in a refractory particle will remain entrained and isolated from isotopic analysis. Where the dissolution of radionuclides from particles is unreliable, the potential to underestimate contamination has been recognised [25].

Measurements of isotopic ratios in environmental samples, such as ^244^Pu/^239^Pu and ^240^Pu/^239^Pu, also enable mass balance arguments [6, 25]. These can be used to determine, for example, the proportion of plutonium arising from a source local to the sample location, as per,

1 $$F_{{L_{239} }} = {\raise0.7ex\hbox{${\left( {R_{240/239} - R_{{G_{240/239} }} } \right)}$} \!\mathord{\left/ {\vphantom {{\left( {R_{240/239} - R_{{G_{240/239} }} } \right)} {\left( {R_{{L_{240/239} }} - R_{{G_{240/239} }} } \right)}}}\right.\kern-0pt} \!\lower0.7ex\hbox{${\left( {R_{{L_{240/239} }} - R_{{G_{240/239} }} } \right)}$}}$$

where: $F_{{L_{239} }}$ is the local proportion of the majority isotope, ^239^Pu, expressed as a fraction of unity; $R_{240/239}$ is the measured ^240^Pu/^239^Pu ratio for a given sample set comprising both local and global contributions. The latter has the isotopic ratio of the global average, $R_{{G_{240/239} }}$, and the former the ratio for the local component, $R_{{L_{240/239} }}$. The local proportion $F_{{L_{239} }}$ must conform with the boundary conditions $0 \le F_{{L_{239} }} \le 1$.

## Experimental

Accelerator mass spectrometry (AMS) [26, 27] is a trace measurement technique in which individual atoms, accelerated from targets derived from samples of interest, are counted. The samples are subject to the preparations referred to above which are described in more detail below. AMS has a high sensitivity to specific charge-to-mass ratios at ultra-trace abundance levels. Measurements are usually made relative to a spike of known abundance (in this context, ^242^Pu) where isobaric interference allows.

### Sampling

Soil samples were collected from Blelham Tarn and Fell Foot on Lake Windermere in the United Kingdom (54.3966° N, 2.9766° S and 54.2130° N, 25,610° S, respectively). Both constitute relatively undisturbed environments, e.g., not subject to regular use by machines etc., with the former registered as a site of special scientific interest: 9 samples were obtained from Blelham Tarn and 15 from Fell Foot on Lake Windermere from the northern and southern shores, respectively, with an aluminium, 50-mm diameter cylindrical extractor of approximately 15 cm in length. The whole of each core, minus surface vegetation, was processed for analysis. Blelham was selected given the potential for comparison with prior art [28] and Fell Foot having not been studied previously in this regard and given its proximity to the Windermere catchment. Each core was transferred to a baking plate for drying in an electric oven set > 100°C for 24 h. Each was then passed through a SWECO Vibro-Energy mill to grind to powder form and subsequently through a sieve of gauge 5 mm to remove material like small pebbles and roots, before radiochemical processing (see below).

### Radiochemical procedures

The samples were divided, with 6 samples subjected to a more rigorous digestion with hydrofluoric (HF) acid and the full set (24 samples) subject to the less aggressive, albeit accepted [29], concentrated nitric acid (HNO_3_) approach, with both described below. The former was used to liberate the actinide content from any refractory component present. Historically, HF-based dissolutions have been used to extract ^244^Pu content only encountered in non-negligible quantities in weapons test fallout entrained in refractory form due to the extreme temperatures in a nuclear detonation. This can be resistant to the latter, less rigorous digestion. Both sets of residues were then subject to separate, trace plutonium measurement with compact AMS.

The samples subject to the HNO_3_ digestion were processed according to Chamizo et al. [29], as follows: 5 g of each sample was weighed and subjected to a generic acid leach followed by iron co-precipitation and removal through ion exchange chromatography. The sample was then put in a Teflon^TM^ beaker and spiked with approximately 3 pg of ^242^Pu with the isotopic ratios from blank samples subtracted from the measured ratios. The plutonium isotopes were extracted from the soil as per Sakaguchi et al*.* [30] with concentrated Suprapur® nitric acid and hydrogen peroxide on a hotplate. The acid solution was centrifuged and filtered three times, followed by evaporation to dryness, then the solution was diluted with 8 M Suprapur® nitric acid. The valences of plutonium were amended using 3 M sodium nitrite before co-precipitation with clean Fe(OH)_3_ to establish full separation of plutonium isotopes. The iron precipitates were re-dissolved in 8 M Suprapur® nitric acid and subject to consecutive removal of plutonium using TEVA® and UTEVA® columns (TrisKem International, France). The eluate, including the plutonium component, was co-precipitated using 1.25 mg of purified Fe^3+^ solution and evaporated to dryness. All plutonium isotopes were converted to the oxide form by heating to 650°C and then combined with 1.3 mg of niobium before being compressed into AMS cathodes. Procedural blanks (n = 24) batched with deionized water were refined in an identical way as the samples and were included in every analytical batch to examine the background influence on the plutonium content. All the samples were evaluated by AMS at the ETH-MILEA AMS facility, Zürich, Switzerland [27].

For the HF digestion, a proportion of each of the samples (approx. 1 g) was transferred into porcelain and dried at 105°C for a minimum of 4 h. The dry masses of the samples were recorded, calcined at 450°C for a minimum of 12 h [31], transferred to PTFE pots and spiked with a ^242^Pu recovery tracer. These were digested using a concentrated HF solution (10 ml) and evaporated to dryness (repeated twice). The dry residue was dissolved in concentrated nitric acid and evaporated to dryness (15 ml, repeated thrice). Following the nitric acid digestion, the solid residue was dissolved in 20 ml of concentrated hydrochloric acid and boiled under cover at approx. 105°C for approx. 1 h. Approximately 1 g of solid boric acid was added to the hot HCl solution, and the solution was allowed to boil for another hour and finally evaporated to dryness.

The solid residue was re-dissolved in 20 ml of 1 M nitric acid, boiled under cover for approximately 1 h and allowed to cool to room temperature. The sample solutions were transferred to 50 ml centrifuge tubes and 10 mg of Fe carrier (as FeCl_3_ solution) was added (to ensure consistency with the blank samples which were without Fe initially). Ammonia solution was added to each sample to form a Fe(OH)_3_ precipitate (pH 8–9). This was separated by centrifuging, the supernatant discarded, and the Fe hydroxide was re-dissolved in HCl to form approx. 25 ml of 9 M HCl solution. To each sample, 0.2 g of sodium nitrite (NaNO_2_, in the form of an aqueous solution, 0.5 ml/sample) was added and the tubes were placed in a hot water bath (ca. 80°C) for approximately 1 h to allow the NaNO_2_ to decompose. Following this step, the samples were allowed to cool to room temperature. A set of anion exchange columns was prepared (1 × 8, 200 mesh, 0.8 × 5 cm) and conditioned with 9 M HCl and loaded with the samples. Once the sample solutions passed the columns, they were washed with 2×20 ml of 9 M HCl, followed by 2×25 ml of 8 M HNO_3_ and again 10 ml of 9 M HCl. All the solutions so far were rejected, and the Pu fraction was eluted to clean beakers using 40 ml of 9 M HCl/ammonium iodide solution. To the precipitate fractions, 5 ml of concentrated nitric acid was added before evaporating to dryness.

The residue was transferred into 1.5 ml Eppendorf vials using 3% HNO_3_ solution spiked with approximately 2–3 mg of Fe in the form of Fe solution (ca. 100 µl of Fe(NO_3_)_3_ solution in water). Ammonia was added to form Fe(OH)_3_ precipitate (0.4 ml of concentrated ammonia solution) and the samples were centrifuged using a micro-centrifuge. The supernatant was rejected, and the precipitate was washed with water and centrifuged again. The washed precipitate was dried at 80°C for a minimum of 4 h, and the dry residue was transferred from the Eppendorf vials to porcelain or quartz crucibles and ignited at 800°C (approx. 100°C/hr ramp was used) for another 4 h to form final Fe_2_O_3_. The produced Fe_2_O_3_ was transferred into clean Eppendorf vials and passed for AMS analysis at the Lancaster MILEA AMS facility, Lancaster, United Kingdom.

## Results

**Fig. 1:**
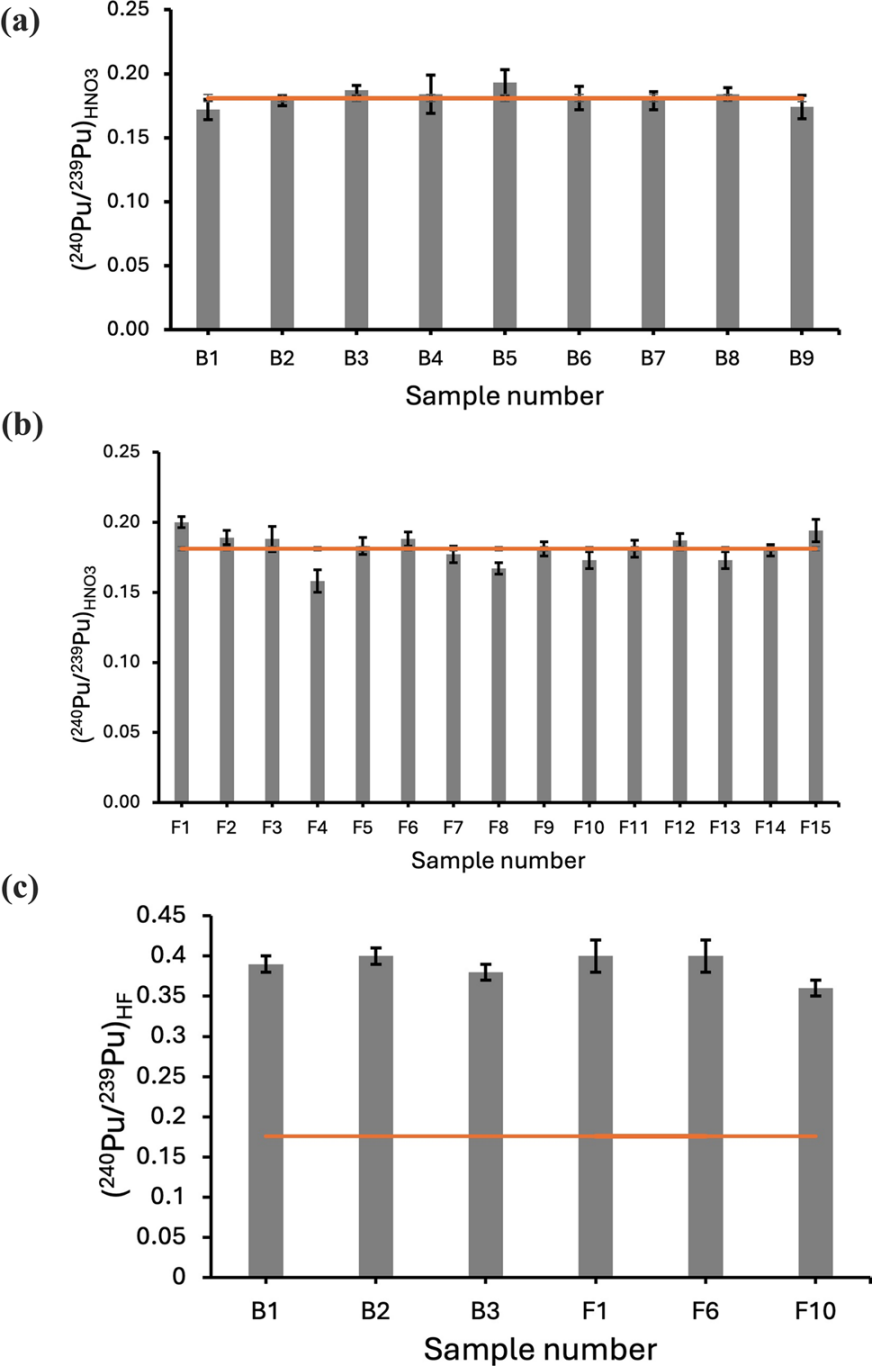
^240^Pu/^239^Pu data for samples processed with HNO_3_ to extract the volatile component a) Blelham and b) Fell foot, and c) for the the samples processed with HF to extract the refractory component, with the global average[8] given by the horizontal lines and the corresponding averages of 0.181 ± 0.002, 0.181 ± 0.003 and 0.390 ± 0.006, respectively

**Fig. 2:**
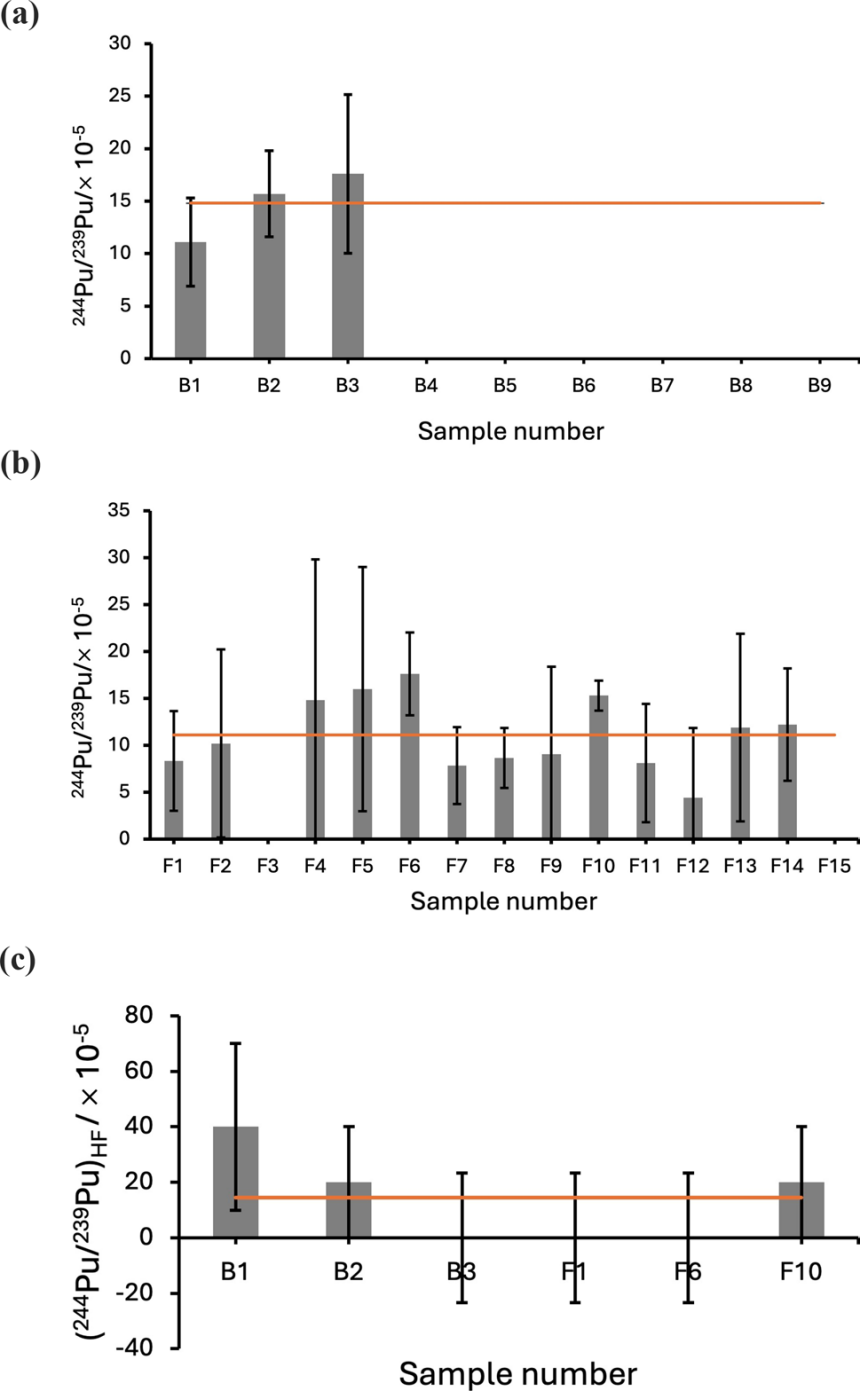
^244^Pu/^239^Pu data for samples processed with HNO_3_ to extract the volatile component a) Blelham and b) Fell foot, and c) for the the samples processed with HF to extract the refractory component, with the global average of (14.4 ± 1.5)×10^–5^ [8] given by the horizontal lines and the corresponding averages of (14.8 ± 0.9)×10^–5^, (11.1 ± 1.0)×10^–5^ and (13.3 ± 6.2)×10^–5^, respectively

The ^240^Pu/^239^Pu data for the samples assessed in this work are given in Fig. 1a, b for the HNO_3_ preparation (Blelham and Fell Foot, respectively). They are combined in Fig. 1c for the HF preparation, and likewise for ^244^Pu/^239^Pu in Fig. 2. The averages arising from these data for both ^244^Pu/^239^Pu and ^240^Pu/^239^Pu are given in Table 1 together with the corresponding global averages and a range of comparison data. For the HF preparation, the average ^244^Pu/^239^Pu across all samples (denoted hereafter (^244^Pu/^239^Pu)_HF_) was measured at (13.3 ± 6.2)×10^–5^ and for the HNO_3_ analysis (^244^Pu/^239^Pu)_HNO3_ = (11.8 ± 1.0)×10^–5^, as per the data in Table 1. These data are consistent with each another, with the global fallout estimate cited above [7] of (14.4 ± 1.5)×10^–5^ and also with measurement of the Pacific ocean [32].

**Table 1:** Measurements for hydrofluoric acid (HF) and nitric acid (HNO_3_) preparations of the corresponding isotopic mass ratios ^240^Pu/^239^Pu and ^244^Pu/^239^Pu, and mass abundances for ^239^Pu, ^240^Pu and ^244^Pu made in this work, compared with prior-art data for the global averages [7–9, 35], Chernobyl within the 30-km zone [11] and estimates of the core [12], Sellafield [36, 37], Bikini/Pacific [32]^*^[33]^**^ and Enewetak [34]

Preparation/measure	Isotopic ratios		Isotopic masses/fg g^−1^		
	^244^Pu/^239^Pu/× 10^–5^	^240^Pu/^239^Pu	^239^Pu	^240^Pu	^244^Pu
HF (refractory)	13.3 ± 6.2	0.390 ± 0.006	201 ± 17	81 ± 8	0.032 ± 0.011
HNO_3_ (volatile)	11.8 ± 1.0	0.181 ± 0.002	203 ± 16	37 ± 3	0.026 ± 0.003
Global averages	14.4 ± 1.5	0.180 ± 0.007	–	–	–
Chernobyl, > 10 km	–	0.408 ± 0.003	–	–	–
Chernobyl, core	–	0.387	–	–	–
Sellafield	< 0.3	0.226 ± 0.001	–	–	–
Bikini atoll^*^	$34.4_{ - 5.9}^{ + 7.7}$	0.253 ± 0.015	–	–	–
Pacific ocean^*^	$16.2_{ - 4.5}^{ + 6.6}$	0.177 ± 0.009	–	–	–
Bikini^**^	28–57	0.31 ± 0.01	–	–	–
Enewetak	118 ± 70	0.363 ± 0.004	–	–	–

However, they are statistically distinct from weapons fallout measurements made with the HF preparation made to isolate the refractory component. Consider, for example, prior measurements made on sediments from Bikini atoll of 34.4 $$\begin{array}{*{20}c} { + 7.7} \\ { - 5.9} \\ \end{array}$$ × 10^–5^ [32] and 28×10^–5^—57×10^–5^ [33], and also for Enewetak of (11.8 ± 0.7)×10^–4^ [34]. Whilst consistent with each other, (^244^Pu/^239^Pu)_HF_ > (^244^Pu/^239^Pu)_HNO3_ confirming the greater degree of ^244^Pu extraction (~ 15%) expected from refractory-entrained fallout with the HF digestion in a mixed volatile/refractory inventory. However, this is largely qualitative, and it is recognised that there are relatively few refractory-processed samples and the errors are large. This difference in ^244^Pu yield, whilst small, agrees with expectations given the Windermere catchment area is some 3500 km from the nearest weapons test site, i.e., Novaya Zemlya, and evidences consistency between the two sets of AMS measurements in the absence of a dedicated ^240^Pu calibration.

**Fig. 3:**
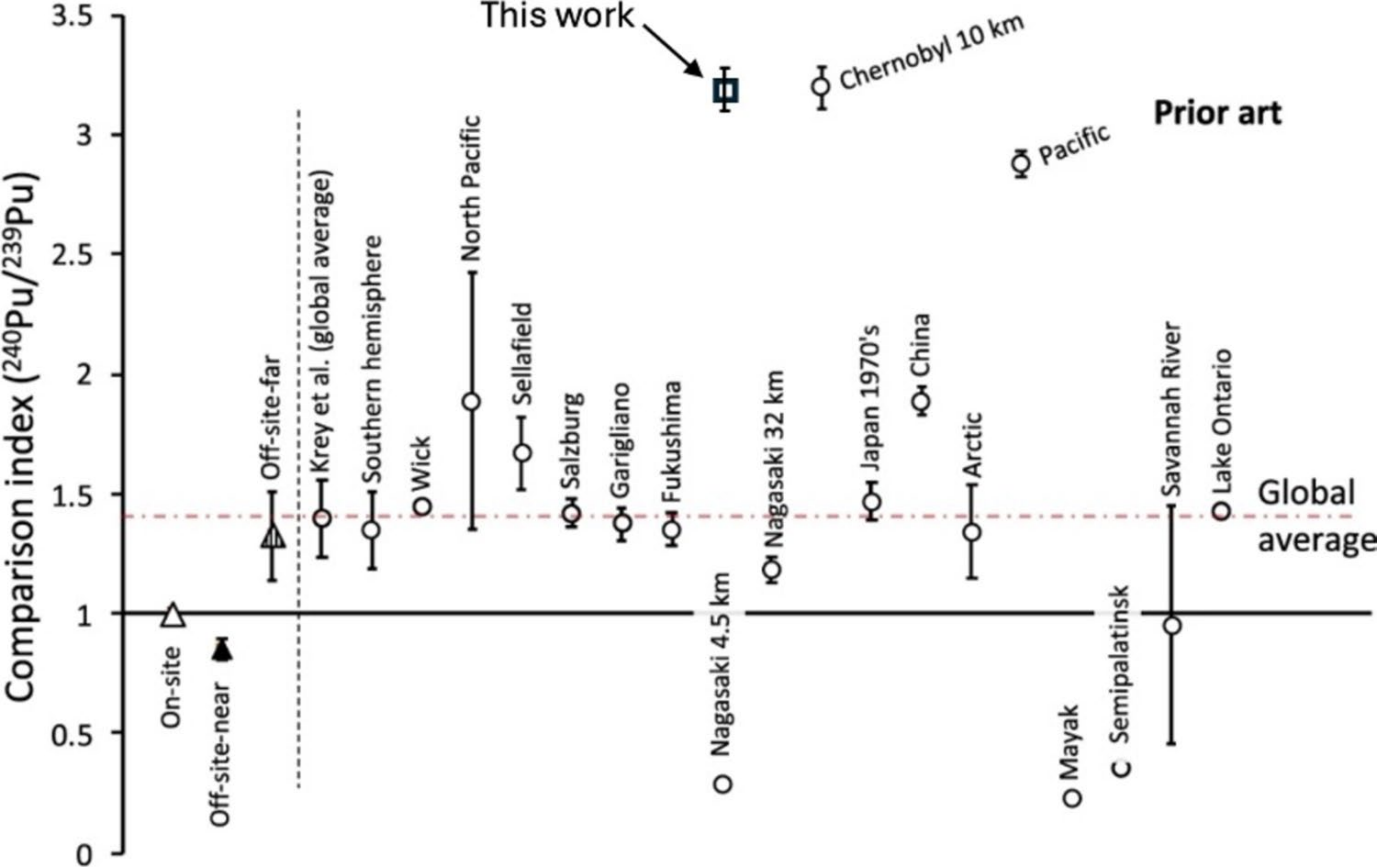
Comparison index for ^240^Pu/^239^Pu ratio defined as the ratios for each of the prior art measurements and that presented in this work, normalised to that measured in samples from on site at Dounreay of ^240^Pu/^239^Pu = 0.123 ± 0.002, annotated from Tighe et al. [6]. Each comparison is identified by its location with references are as per Tighe et al. and uncertainties derive from the original works. The datum for this work is as indicated 3.17 ± 0.07 consistent with that calculated for the prior art of 3.31 ± 0.06 for measurements made of samples > 10 km distant from Chernobyl [16]

Similarly, across all samples (^240^Pu/^239^Pu)_HNO3_ = 0.181 ± 0.002 which is consistent with measurements of the global average[8, 9, 35] and the Pacific ocean [32] whereas we obtain (^240^Pu/^239^Pu)_HF_ = 0.390 ± 0.006. This is inconsistent with the global average, implying high levels of mixing with global fallout by a component having a much more significant proportion of ^240^Pu in the refractory component. In Fig. 3, the (^240^Pu/^239^Pu)_HF_ data are plotted as a comparison index, defined as the datum defined by the corresponding measurement made for Dounreay [6]. This figure is an annoted version of the related figure from the cited work and includes a variety of data for other measurements and for Chernobyl [16], highlighting this consistency.

## Discussion

For the ^240^Pu-rich material responsible for the high (^240^Pu/^239^Pu)_HF_ to have originated from Sellafield, some 60 km distant from the Windermere catchment area, would imply either the Windscale accident [38], Magnox material [11] arising in permitting discharges [39] or that originating more widely from Sellafield activities [36, 37] as candidate sources. These are characterised by local ^240^Pu/^239^Pu ratios, $R_{{L_{240/239} }} ,$ of 0.0218, 0.23, and (0.226 ± 0.001), respectively. Windscale was designed to produce military plutonium thereby having a typical $R_{{L_{240/239} }}$ < 0.07. The accident was a fire rather than a prompt excursion and is considered to have had little or no lasting radiological significance nearby [40]. However, this has rarely been probed to date in terms of whether there could be an associated trace-level refractory phase in the fallout associated with it. On the other hand, it is noted that even those weapons tests returning relatively significant ^240^Pu levels and where the necessary digestion has been applied tend to be of order (^240^Pu/^239^Pu)_HF_ ~ (0.253 ± 0.015) [32] and hence all are unlikely sources on this basis.

Recognising that the sources postulated above would also need to have involved temperatures sufficient to yield the refractory bias we observe, mass balance considerations made by substituting $R_{240/239} =$ (^240^Pu/^239^Pu)_HF_ for each of these options into Eq. (1) with $R_{{G_{240/239} }}$ = (0.180 ± 0.007) [9] yields unphysical estimates for the proportion of ^239^Pu being deposited by any of these sources. Rather, this implies either $F_{{L_{239} }}$ < 0 (Windscale) or $F_{{L_{239} }}$ > 1 (Magnox and Sellafield) because the ^240^Pu proportion in $R_{{L_{240/239} }}$ for these possibilities is too small at < 23%. The requirement highlighted above, that $0 \le F_{{L_{239} }} \le 1$ corresponds to either $F_{{L_{239} }} = 1$, with all plutonium in a sample being local in origin, i.e., $R_{{L_{240/239} }} = R_{240/239}$ or, conversely, $F_{{L_{239} }} = 0$ and all plutonium being of global origin, as per $R_{{L_{240/239} }} = R_{{G_{240/239} }}$. The number of known scenarios that might yield the localised contribution necessary to give $R_{{L_{240/239} }} \ge$(0.390 ± 0.006), and which avoid $F_{{L_{239} }} > 1$, is therefore limited. Prior analysis of the field [6] suggests only ^240^Pu/^239^Pu levels associated with Chernobyl [16] and the Pacific [37] fall into this category, where the latter is unlikely to be a *local* contributor to material being some 12,500 km distant from the sample site, as opposed to 2,250 km for Chernobyl.

However, on the basis of prior caesium analysis it is known that a significant source of fallout at trace levels in the Windermere catchment [28, 41] is from Chernobyl. ^240^Pu/^239^Pu was measured with inductively coupled plasma mass spectrometry (ICPMS) in soil samples in the 30-km area around Chernobyl [11] after the associated accident (in which it is estimated that ~ 8.7×10^13^ Bq ^239+240^Pu were released [42]). These suggest a value of ^240^Pu/^239^Pu = 0.410 ± 0.002 for the sampling site nearest (6 km) the plant with the highest ^239+240^Pu concentration. Earlier estimates [17] by the former Soviet Union and the UK suggested a range 0.38 to 0.56, respectively. The lower of these agrees with estimates of the reactor inventory at the time of the accident [12, 13].

Rather than being limited to two components in the mass balance analysis corresponding to the local and global contributions (as per Eq. 1 [6]), three component ratios can be defined for this specific hypothesis for global, Chernobyl and Sellafield contributions defined by ^240^Pu/^239^Pu in each case, i.e., $R_{{G_{240} }}$, $R_{{C_{240} }}$ and $R_{{S_{240} }}$, respectively. Equation 1 can then be repurposed for ^240^Pu and ^244^Pu to yield two simultaneous equations which when solved give the expression for the Chernobyl component of ^239^Pu, $F_{{C_{239} }}$, as per Eq. 2 below. A full derivation is given in the supplementary material.

2 $$F_{{C_{239} }} = {{\left[ {\frac{{R_{{\frac{240}{{239}}}} - R_{{G_{240} }} }}{{R_{{S_{240} }} - R_{{G_{240} }} }} + \frac{{R_{{G_{244} }} - R_{{\frac{244}{{239}}}} }}{{R_{{S_{244} }} - R_{{G_{244} }} }}} \right]} \mathord{\left/ {\vphantom {{\left[ {\frac{{R_{{\frac{240}{{239}}}} - R_{{G_{240} }} }}{{R_{{S_{240} }} - R_{{G_{240} }} }} + \frac{{R_{{G_{244} }} - R_{{\frac{244}{{239}}}} }}{{R_{{S_{244} }} - R_{{G_{244} }} }}} \right]} {\left[ {\frac{{\left( {R_{{C_{240} }} - R_{{G_{240} }} } \right)}}{{R_{{S_{240} }} - R_{{G_{240} }} }} - \frac{{\left( {R_{{C_{244} }} - R_{{G_{244} }} } \right)}}{{R_{{S_{244} }} - R_{{G_{244} }} }}} \right]}}} \right. \kern-0pt} {\left[ {\frac{{\left( {R_{{C_{240} }} - R_{{G_{240} }} } \right)}}{{R_{{S_{240} }} - R_{{G_{240} }} }} - \frac{{\left( {R_{{C_{244} }} - R_{{G_{244} }} } \right)}}{{R_{{S_{244} }} - R_{{G_{244} }} }}} \right]}}$$

Substituting for the range in $R_{{C_{240} }}$ above, i.e., 0.38 $\le R_{{C_{240} }} \le$ 0.56 yields a corresponding range in Chernobyl-derived ^239^Pu, as follows (61 $\pm 15$)% $\le F_{{C_{239} }} \le$ (126 $\pm 35$)%. The average of this range, $\overline{{R_{{C_{240} }} }}$ = 0.47 ± 0.01[11, 42] corresponding to $\overline{{F_{{C_{239} }} }}$ = (83 $\pm 21$)%. This is a physically consistent estimate for $F_{{C_{239} }}$ and, whilst high, concurs qualitatively with prior reports highlighted earlier based on the caesium analysis that implied most fallout in the Windermere catchment to be from Chernobyl [28, 41]. This suggests the presence of a significant refractory component requiring temperatures for formation consistent with a nuclear conflagration and the observed blue flash, dispersed at distances consistent with an atmospheric injection and having a Chernobyl-signature level of ^240^Pu.

### Implications for atmospheric dispersion

Reports of radionuclide distribution and migration from Chernobyl are extensive but have often been made (not unreasonably) to assess radionuclide danger and hence with radiation spectroscopy apparatus. This requires sufficient radioactivity [34] to enable airborne surveys and often the use of handheld apparatus etc. These factors, along with the assumptions of relatively low temperatures incapable of volatising refractory elements such as plutonium and low altitudes associated with the fire-driven dispersion, imply plutonium fallout limited to a 30-km zone [43] albeit in significant amounts, i.e., > 1 GBq km^−2^.

For activity concentrations below that given above, it is feasible that radioactivity techniques would have been insensitive to trace amounts of refractory-borne plutonium. Further, for the plutonium isotopes of interest, the γ-ray signatures are likely to have been limited in intensity and for the sequelae of some, i.e., ^241^Am, are also at energies where detection efficiencies are very low. The conventional method of α-spectroscopy may have been impeded by the micron-size particulate debris attenuating the number of α particles reaching a detector. Forensic analysis at levels below such conventional methods, such as AMS, on samples prepared to penetrate refractory content has the potential to probe a submicron component formed at very high temperature and compatible with long-range transport aided by an atmospheric injection. Whilst such material poses little danger radiologically, it constitutes forensic evidence for a different primary mechanism of dispersion which would have preceded that which resulted in a longer-term, low-altitude dispersion of significant quantities of radioactivity near to the site of the accident.

### Prompt fission excursion in a low-enriched reactor matrix

**Fig. 4:**
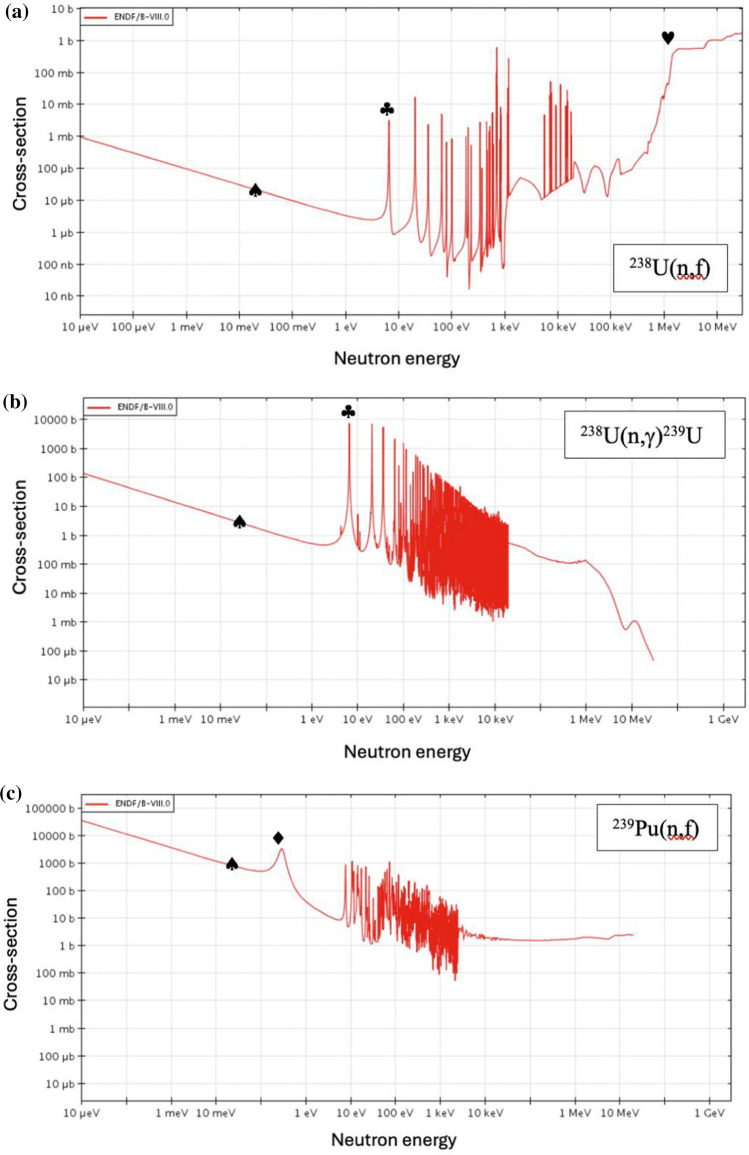

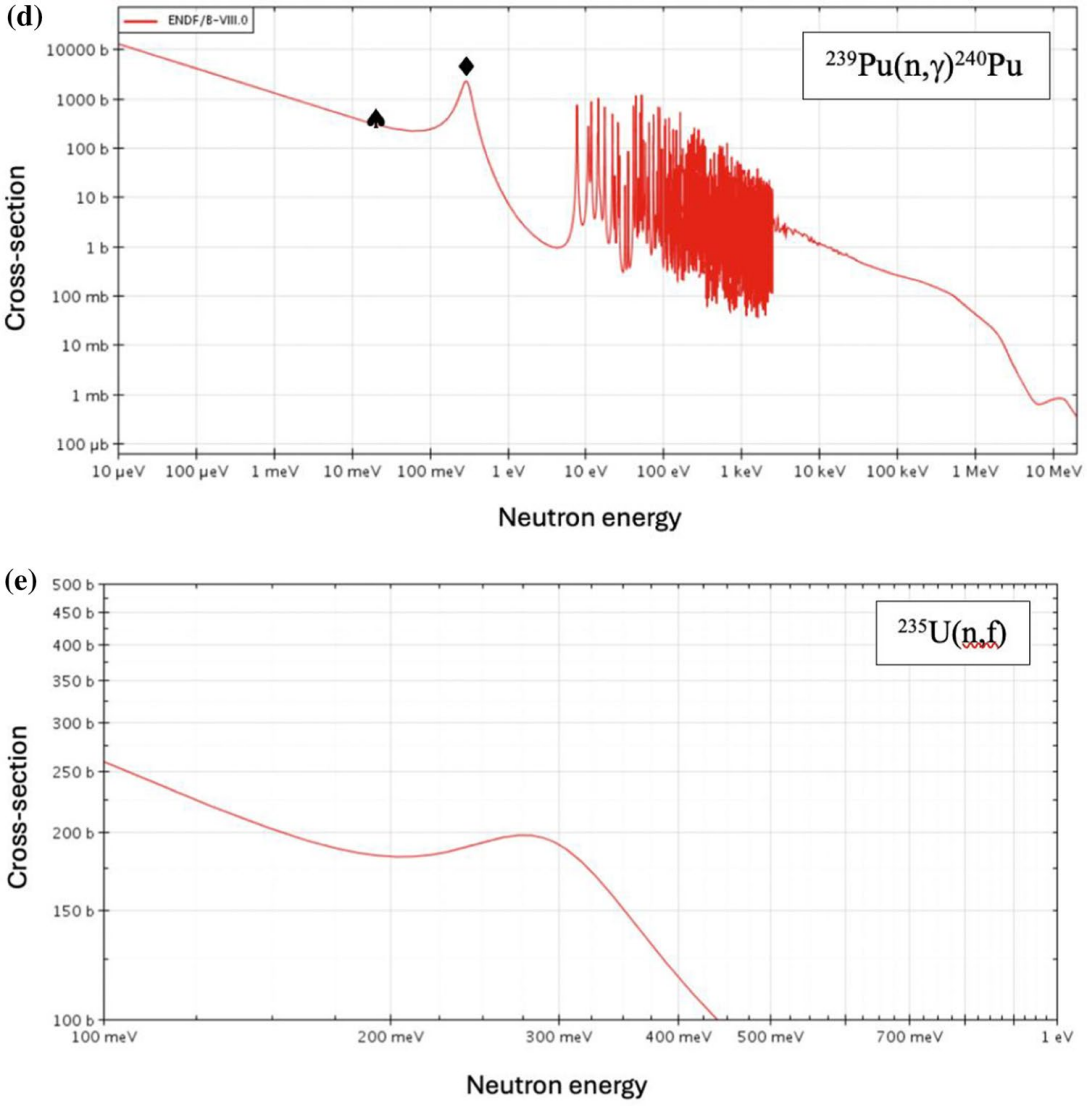
Microscopic cross-sections as a function of neutron energy (ENDF/B-VIII.0, www.oecd-nea.org/janisweb) for a) ^238^U(n,f), b) ^238^U(n,γ)^239^U, c) ^239^Pu(n,f), d) ^239^Pu(n,γ)^240^Pu and e) for the 0.1–1 eV energy range in ^235^U(n,f), showing the 6.7 eV onset of resonances (♣), the 0.3 eV maximum (♦), the ~ 1 MeV threshold for fast fission (♥) and thermal energies (♠). The ^238^U resonances and fast fission threshold are unachievable at temperatures feasible for the reactor environment prior to the full progression of the accident but the S-wave resonance at 0.3 eV for.^239^Pu lies in the temperature range consistent with prior reports [2, 4]

For a rapid excursion to have been feasible in a uranium power reactor, the fissionable atom number density $n$, neutron spectrum $E_{n}$ and/or flux $\phi$ would need to have increased significantly above normal, steady-state operational levels, albeit only locally in a small number of fuel channels. Unit 4 at Chernobyl used a graphite moderator and 192 t uranium dioxide (UO_2_) fuel [1] enriched to 2% ^235^U and was at an average of ~ 1.1% across the core at the point of accident, ranging from 1.9–0.84%. It has been estimated that approximately 450 kg of ^239^Pu had accumulated in the fuel at the time of the accident [12]. The fuel was contained in pressure tubes through which light water coolant flowed which was allowed to boil in normal operation. A thermal neutron spectrum is exploited to sustain a fission chain reaction predominantly in ^235^U, with smaller contributions from ^238^U (fast fission) and ^239^Pu. A period of operation is needed for the latter to breed in from the former, noting as above that Unit 4 was in a relatively high burnup state at the time of the accident. The proportion of fast fission in ^238^U is anticipated to have been small relative to the other contributors because the proportion of neutrons with sufficient energy, i.e., $E_{n}$ > 1 MeV will have been small, i.e., a fast fission factor ε ~ 1.02 given the ^238^U(n,f) cross-section dependence with energy given in Fig. 4a. The influence of inelastic scattering on ^238^U as opposed to fast fission will have acted to soften fission neutron spectra, hence reducing their ability to cause fast fission.

However, with the coolant water vaporised by the sudden power increase, the latter in part a result of water being displaced by the graphite followers inserted along with their associated control rods, two neutronic factors are relevant:

- Firstly, the sink for neutrons via absorption on hydrogen in water is lost and neutron economy increases and,
- Secondly, positive void feedback due to the falling moderator-to-fuel ratio acts to increase reactivity and to harden the neutron spectrum.

Together, these factors result in more neutrons with higher energies albeit transiently, potentially buoyed further by the higher average number of neutrons emitted per fission [44] from fast fission in ^235^U, ^239^Pu and ^238^U, compared to thermal (cf., $\nu_{{238{\mathrm{\_fast}}}}$ = 2.82 ± 0.02 as per $\nu_{{235{\mathrm{\_thermal}}}}$ = 2.436 ± 0.002).

These factors notwithstanding, a greater, harder neutron population would be necessary to overcome moderation by inelastic scattering on ^238^U and hence the quenching of fast fission in ^238^U. This would also be compromised by Doppler broadening of ^238^U(n,γ)^239^U resonances with escalating temperature, as per negative fuel temperature feedback. Similarly, despite its higher cross-section relative to fast fission for $E_{n}$ > 6.7 eV (see Fig. 4b), the potential for the ^238^U neutron capture pathway to augment the overall fission yield ultimately via its yield of ^239^Pu would likely be stalled temporally by the relatively long half-lives of the intermediary isotopes ^239^U and ^239^Np, compared to the timescales associated with a nuclear excursion. It therefore appears unlikely that the higher-energy neutron flux arising from a rapid fall in moderator-fuel ratio would have been sufficient to have acceded to the favourable cross-sections for ^238^U(n,f) for $E_{n}$ > 1 MeV, despite the higher ^238^U number density, i.e., ×  ~ 100 compared to that of ^235^U at ~ 1%. Conversely, the ambient temperatures necessary to provoke this otherwise are beyond what is feasible notwithstanding the scale of the catastrophe, i.e., > 80,000 K, given the first fast fission resonances in ^238^U arise for $E_{n}$ > 6.7 keV.

By contrast, the microscopic cross-sections [45] for both ^239^Pu(n,f) and ^239^Pu(n,γ)^240^Pu are favoured over thermal by nearly an order of magnitude at ~ 0.3 eV, cf., $\sigma_{f}$ ~ 3250 b compared to ~ 750 b (see Fig. 4c) and $\sigma_{c}$ ~ 2250 b and 275 b, respectively (see Fig. 4d) because of the broad S-wave resonance at this point. Further, broadening due to escalating temperature not only suggests enhanced ^240^Pu production but also ^239^Pu consumption by fission in a zone of fuel so affected to cause an escalation in temperatures necessary to render this feasible, i.e., of between 1200 K < $T$ < 12,000 K which corresponds to the region of the favoured cross-section, 0.1 eV < $kT$ < 1 eV. This is consistent with the refractory thresholds highlighted earlier and inferences of the black body radiation of cooling debris associated with the blue flash [4], $T$ ~ 7000 K. Further this scenario has the potential for ^240^Pu production *and* ^239^Pu consumption in a zone of material party to such temperatures. With the scenario that this was ejected by the action of the excursion, this is consistent with (^240^Pu/^239^Pu)_HF_ being above that found in the volatile component deposited locally, as per (^240^Pu/^239^Pu)_HNO3_. It is noted that new transmission measurements of the 0.3 eV resonance have been recommended recently [46].

The ^235^U enrichment in fuel assemblies subject to the greatest burnup has been estimated to be 0.84% [12] and the ^239^Pu composition ~ 0.25%, implying a similar number of ^235^U atoms as ^239^Pu atoms at the time of the accident, $n$(^235^U) ~ 3.4×$n$(^239^Pu). Whilst there is also a corresponding resonance at 0.3 eV for ^235^U(n,f), this peaks [45] at a much lower cross-section than ^239^Pu, cf., 200 b as opposed to ~ 3250 b for ^239^Pu(n,f), see Fig. 4e. This supports the focus here on plutonium as a key factor in this hypothesis.

In moving from thermal to the 0.1 eV range, the capture-to-fission ratios, α, for ^235^U and ^239^Pu increase by a similar factor of ~ 1.5–1.9 to one another because the neutron capture cross-section increases relative to that for fission. This suggests an overall reduction in the propensity for fission. However, the effect of the 0.3 eV resonance in, say moving from ~ 0.1 eV to its peak is insightful since for ^235^U (see Fig. 4e) this results in a decrease in α of only ~ 10% compared to 60% for ^239^Pu. This suggests a significant enhancement in fission potential for the latter isotope. In short, the increase in fission and capture cross-section for temperatures enabling neutron energies corresponding to $kT$ ~ 0.3 eV is greater for ^239^Pu than for ^235^U. This fission pathway contributes to the possibility of an excursion and the consumption of ^239^Pu, whilst the capture pathway would exacerbate ^240^Pu yield. Taken together both are consistent with there being a relatively high ^240^Pu/^239^Pu signature entrained in refractory fallout dispersed at high altitude.

In summary, vaporisation of the coolant at a point when reactivity was already increasing rapidly do to the complete withdrawal of control rods is likely to have i) increased the reproduction factor ($\eta$) and thermal utilisation factor ($f$) due to enhanced ^239^Pu fission, and ii) increased the resonance escape probability ($p$) and fast fission factor ($\varepsilon$) due to the hardened neutron spectrum. All would contribute to a rapid increase in multiplication factor ($k_{\infty }$), which would manifest as an enhancement in ^240^Pu production and ^239^Pu consumption in a localised, affected zone, consistent with the high ^240^Pu/^239^Pu observed.

## Conclusions

The observation of a relatively high ^240^Pu/^239^Pu ratio in refractory fallout appearing at long range based on nearest-neighbour isotopics (in this case measurements made with refractory-resistant preparations taken close to Chernobyl), relative to what is known locally, suggests the potential for two fallout mechanisms to be at work: one at temperatures that promote comprehensive radionuclide mixing whilst the associated carrier material is molten, and the other at lower temperatures where the radioactive material is adhered to the surface to already-solidified, carrier particles.

The RBMK reactor design comprises UO_2_ fuel in zircaloy cladding, inserted in zircaloy pressure tubes with stainless steel headers and footers, surrounded by a nuclear graphite moderator with boron carbide control rods. The relevant corresponding melting points are 2865°C (UO_2_), 1850°C (Zr), ~ 1500°C (Fe), 3600°C (graphite) and 2350°C (B_4_C). The requirement that, for refractory fallout, the carrier material has a melting point lower than the radioactive debris entrained within it, and the proximity of these materials to the fuel, implies they are most likely to have been consumed first in the destruction following a rapid excursion. This suggests, for example, a carrier comprised primarily of zirconium with iron, boron and carbon present in lesser quantities. It also agrees with observations of micron-sized, hot particles comprised of iron, zirconium and silicate compounds formed during fuel melting found in fallout beyond the former USSR [12]. At the time of the accident, Unit 4 had operated for 865 days at a capacity load factor of 0.82 and ~ 75% of fuel assemblies being first load bundles with an average burn-up over the core of 10.9 MWd/kg [12].

At relatively long timescales after the accident, the reactor top plate was observed to glow yellow–red [47] implying a temperature range of the fire following the destruction of the reactor of order 770–1250°C. This would be too low to melt any of the fuel components but sufficient to disperse solidified carrier debris with radioactive products adhered to the surface of the constituent particles, i.e., the volatile phase. This might be expected to have peaked locally, as per the measurements made prior to this work referred to above [11, 16] and the known focus of the debris dispersed by the fire.

However, prior to the destruction of the reactor, an uncontrolled excursion comprising neutron-stimulated exothermic reactions appears likely to have initiated the accident [48].Whilst characterised by temperatures and pressures lower than in a weapon, some of the fuel becoming prompt critical may have been sufficient to increase the neutron flux sufficiently rapidly for a proportion of it to be dispersed energetically, given temperatures of 6000–7000°C were reached, and for the plutonium isotopic vectors to be changed in this material as observed relative to the preceding, normal period of operation. Based on the energy estimated from the diffusion of fission products in the graphite associated with plugs in the reflector, temperatures of up to 40,000°C have been regarded as feasible, which is also supported by dust evident throughout the plant as a result of the vaporization of fuel and graphite. These temperatures are also consistent with the black-body emission cited as responsible for the blue flash [3] observed shortly after the explosions (as the heated vaporized material cooled in the atmosphere above the plant), and also the long-range deposition in northern England consistent with an atmospheric injection of refractory debris.

Concerning the Windscale hypothesis, the ^240^Pu/^239^Pu ratio for Windscale debris likely to have been much lower [38] because the plant made material favouring a low ^240^Pu abundance. Also, the reactor in this case was fuelled with metal uranium with a boiling point higher than uranium dioxide, at 4131°C, and with the aluminium clad burning > 1000°C with graphite at ~ 800°C implying the uranium would oxidise at much lower temperatures. Consequently, it appears likely the majority was dispersed in volatile rather than refractory form. This is supported by the prior analysis [28] mentioned above of Blelham Tarn sediments suggesting a significant proportion of the ^137^Cs fallout in this area derived from Chernobyl; this not being reflected in the associated plutonium data may also be due to α-counting procedures [41] not able to penetrate a significant refractory component.

As for other studies of the resilient, refractory ‘glassy’ component, as mentioned, the difference between weapons fallout and dilute sedimentary deposits has been distinguished via ^244^Pu/^239^Pu ratios [32] and the distinction in uptake of ^137^Cs and Pu from insoluble, glassy particles has been observed. Further, ^240^Pu/^239^Pu ratios consistent with Nagasaki fallout and dispersed fallout (low and consistent with the global average, respectively) have been observed [49]. These contrast with elevated levels (0.293 ± 0.012) observed in a concentrated source in organic detritus from a rooftop drainage system in use at the time of the Fukushima accident, which is consistent with reactor material but lower in ^240^Pu than for Chernobyl material, as expected.

For the ^240^Pu/^239^Pu component measured previously in samples 6 km from the plant (Muramatsu, 2000), a nitric acid preparation was used. We hypothesise that this would have introduced bias towards the volatile component spread by the force of the chemical explosion(s) and the fire that followed, rather than being vaporised. Similarly, an atmospheric injection is unlikely to have deposited refractory material formed in the heat from a nuclear excursion close to the plant, with the potential for a volatile component to be deposited nearby and a refractory component further afield. That work [16] also reports relatively high ^137^Cs/^239+240^Pu ratios consistent with the refractory proportion of ^239+240^Pu being under-extracted and hence returning an artificially low measurement via ICPMS.

Often in nuclear weapons tests, the refractory component can be concentrated near to the site of detonation associated with the zone in which the temperature is sufficiently high whereas the cooling further afield results in temperatures [23] below the reference temperature at which it is incorporated as a volatile. By contrast, at Chernobyl the temperature was lower and hence debris may have cooled more quickly, condensing earlier, prior to injection into the atmosphere by chemical reactions, with the fire dispersing fuel debris locally and in volatile form after that ejected by the nuclear excursion.

Whilst many reports have been made concerning Chernobyl fallout, unanimity remains lacking concerning radionuclide release magnitudes and dynamics during the accident [47]. Where analysis of fuel particles in soil samples local to the plant has been attempted, the preparations used (i.e., boiling in concentrated HNO_3_) may not have been sufficient to mobilise refractory-bound deposits. Hence, they risk returning ^240^Pu/^239^Pu largely consistent with global fallout and much lower Pu contributions from Chernobyl, cf., [50] albeit at higher latitudes. Conversely, the use of the more rigorous preparations necessary to liberate refractory deposits has not been widespread. Further, assessment has often been via conventional radioactivity-based methods. It is doubtful whether these will have discerned trace levels spread beyond the immediate 30-km radius. Further they can be reliant on the higher specific activity Pu signatures such as ^238^Pu rather than the more analytically challenging ^239,240^Pu.

Usually for plutonium isotopics a ^242^Pu spike is utilised as a calibration and the associated ratios are derived from this, as in this work. However, it is recognised that an additional calibration using a ^240^Pu standard would provide further independent evidence of the ^240^Pu excess reported in this work. This is planned for the future.

## Supplementary Information

Below is the link to the electronic supplementary material.

Supplementary material 1 (DOCX 78 kb)
