# Supplementary material for: Plutonium signatures in refractory fallout support a Chernobyl nuclear jet hypothesis
Source: J Radioanal Nucl Chem. 2025 Nov 20;334(12):9287–99. doi: 10.1007/s10967-025-10541-0 (PMC12827445; doi:10.1007/s10967-025-10541-0)
Supplement: Supplementary file 1 — Supplementary material 1 (DOCX 78 kb) [file 10967_2025_10541_MOESM1_ESM.docx]

Supplementary information

**Data**

|  | F1 | F2 | F3 | F4 | F5 | F6 | F7 | F8 | F9 | F10 | F11 | F12 | F13 | F14 | F15 |
| --- | --- | --- | --- | --- | --- | --- | --- | --- | --- | --- | --- | --- | --- | --- | --- |
| HNO_3_ | 0.200 | 0.189 | 0.188 | 0.158 | 0.183 | 0.188 | 0.177 | 0.167 | 0.181 | 0.173 | 0.181 | 0.187 | 0.173 | 0.180 | 0.194 |
| ±σ | 0.004 | 0.005 | 0.009 | 0.008 | 0.006 | 0.005 | 0.006 | 0.004 | 0.005 | 0.006 | 0.006 | 0.005 | 0.006 | 0.004 | 0.008 |
| HF | 0.40 | - | - | - | - | 0.40 | - | - | - | 0.36 | - | - | - | - | - |
| ±σ | 0.02 | - | - | - | - | 0.02 | - | - | - | 0.01 | - | - | - | - | - |

Table S1 Fell Foot ^240^Pu/^239^Pu data for the HNO_3_ and HF preparations, average (^240^Pu/^239^Pu)_HNO3_ = 0.181± 0.002, (^240^Pu/^239^Pu)_HF_ = 0.39± 0.01.

Table S2 Blelham ^240^Pu/^239^Pu data for the HNO_3_ and HF preparations, average (^240^Pu/^239^Pu)_HNO3_ = 0.181± 0.003, (^240^Pu/^239^Pu)_HF_ = 0.390± 0.006.

|  | B1 | B2 | B3 | B4 | B5 | B6 | B7 | B8 | B9 | Average |
| --- | --- | --- | --- | --- | --- | --- | --- | --- | --- | --- |
| HNO_3_ | 0.172 | 0.179 | 0.187 | 0.184 | 0.193 | 0.181 | 0.179 | 0.184 | 0.174 | 0.181 |
| ±σ | 0.008 | 0.004 | 0.004 | 0.015 | 0.010 | 0.009 | 0.007 | 0.005 | 0.009 | 0.003 |
| HF | 0.39 | 0.40 | 0.38 | - | - | - | - | - | - | 0.390 |
| ±σ | 0.01 | 0.01 | 0.01 | - | - | - | - | - | - | 0.006 |

Data correction and normalisation

Assuming a perfectly pure ^242^Pu solution and perfect tuning of the AMS, there would be no counts measured on the blank target other than ^242^Pu. As it is, false counts (i.e., counts not from the sample) can arise from: 1) other Pu isotopes present as contaminants within the spike solution, 2) the upper $E/q$ ^238^U tail from beamline contamination impinging on the ^239^Pu measurement (51) (52) (27), and 3) other ions in the $m/q$ ratio 80/+1 entering the GIC and overlapping in the MCA with ^240^Pu measured in the 3+ charge state. To account for both impurities and ion-optics "crosstalk", a blank measurement is usually taken to quantify the amount of background relative to the spike isotope, which is then used to correct each of the measured n(^2XX^Pu)/n(^242^Pu) ratios for the samples.

Isotope ratios for the HNO_3_ preparation were measured in this manner, using spike blanks containing ^242^Pu-spiked iron oxide carrier and Nb powder; niobium blanks containing only Nb powder; and Pu isotope ratio standards. However, the HF preparation ratios were measured with spike blanks only, and the IAEA-326 standard material used for tuning the beam optics for Pu is certified for alpha activity rather than isotope ratio (IAEA-326 Certificate). This means that the blank correction method used for the HNO_3_ data cannot be replicated directly for the HF data.

Instead, three different methods for correcting the HF measurements were considered: (A) a ratio correction using the spike blank; (B) a rate correction using the spike blank; and (C) a proportional correction following a model of isotope contaminants within different purities of ^242^Pu spike (53). The HNO_3_ preparation yielded far greater counts on target per mass of sample than the HF preparation, possibly due to a valency-loss of sample Pu during ion exchange separation (54). The measured Pu isotope mass abundances for the HF preparation were therefore efficiency-corrected by normalising the alpha activity calculated from the measured mass abundance of the IAEA-326 target to the certified value.

Correction (A) assumes that all blank counts are due to the ^242^Pu-spiked iron oxide carrier and Nb powder in the targets, providing a background ratio that is then subtracted from all sample measurements. This gives a n(^240^Pu)/n(^239^Pu) measurement of 0.50±0.02 with an efficiency of (35±2)%. Correction (B) assumes that all blank counts are due to beamline contamination and crosstalk, subtracting the blank count rate for each isotope as background from the measured count rate. This gives a n(^240^Pu)/n(^239^Pu) measurement of 0.458 ± 0.010 with an efficiency of (41±3)%. Correction (C) applies a proportional correction to each isotope count based on the amount of contaminant expected in different purities of ^242^Pu spike solution (Engle, 2013). This gave a n(^240^Pu)/n(^239^Pu) measurement of 0.395±0.007 for 95% purity ^242^Pu and 0.405±0.007 for 99% purity ^242^Pu, or a final average of 0.400±0.005 with an efficiency of (44±2)%. Being the most comprehensive method, the averages obtained via correction C were selected and have been used in the main body of the work above.

Fig. S1 Pu isotope mass fractions considered for different purities of ^242^Pu spike (53)

Table S3 Data for the purposes of comparison of the different normalisation methods

| Preparation/ measure | Isotopic ratios (n/n) | | Isotopic masses/fg g^-1^ | | | Total efficiency (%) |
| --- | --- | --- | --- | --- | --- | --- |
|  | ^244^Pu/^239^Pu / ×10^-5^ | ^240^Pu/^239^Pu | ^239^Pu | ^240^Pu | ^244^Pu |  |
| (A) HF | 13.4±6.4 | 0.503±0.017 | 215±32 | 107±14 | 0.042±0.021 | 35±2 |
| (B) HF | 14.6±7.0 | 0.458±0.010 | 200±27 | 91±12 | 0.036±0.018 | 41±3 |
| (C, 99%) HF | 13.4±6.4 | 0.405±0.007 | 195±23 | 80±10 | 0.032±0.016 | 47±3 |
| (C, 95%) HF | 13.0±6.4 | 0.395±0.007 | 207±25 | 83±11 | 0.033±0.017 | 42±3 |
| (C, avg.) HF | 13.2±4.5 | 0.400±0.005 | 201±17 | 81±8 | 0.032±0.011 | 44±2 |
| HNO_3_ | 11.8±1.0 | 0.181±0.002 | 203±16 | 37±3 | 0.026±0.003 |  |
| Global averages | 14.4±1.5 | 0.180±0.007 | - | - | - |  |

Mass balance with three components

For the case of there being three components to the fallout, the measured ratio $R_{240/239}={{}^{240}\text{Pu}}/{{}^{239}\text{Pu}}$, can be written,

$R_{240/239}=\frac{m_{G_{240}}+m_{C_{240}}+m_{S_{240}}}{m_{G_{239}}+m_{C_{239}}+m_{S_{239}}}$ (S1)

where $m_{G_{240}}$, $m_{C_{240}}$ and $m_{S_{240}}$are the mass of ^240^Pu for the global, Chernobyl and Sellafield contributions, respectively, and likewise for ^239^Pu as per $m_{G_{239}}$, $m_{C_{239}}$ and $m_{S_{239}}$. S1 can be written,

$$R_{240/239}=\frac{m_{G_{240}}}{m_{G_{239}}+m_{C_{239}}+m_{S_{239}}}+\frac{m_{C_{240}}}{m_{G_{239}}+m_{C_{239}}+m_{S_{239}}}+\frac{m_{S_{240}}}{m_{G_{239}}+m_{C_{239}}+m_{S_{239}}}$$

$$=\frac{m_{G_{240}}}{m_{G_{239}}}\frac{1}{\left(1+m_{C_{239}}/m_{G_{239}}+m_{S_{239}}/m_{G_{239}}\right)}+\frac{m_{C_{240}}}{m_{C_{239}}}\frac{1}{(m_{G_{239}}/m_{C_{239}}+1+m_{S_{239}}/m_{C_{239}})}+\frac{m_{S_{240}}}{m_{S_{239}}}\frac{1}{(m_{G_{239}}/m_{S_{239}}+m_{C_{239}}/m_{S_{239}}+1)}$$

$$=R_{G_{240}}\frac{1}{\left(1+m_{C_{239}}/m_{G_{239}}+m_{S_{239}}/m_{G_{239}}\right)}+R_{C_{240}}\frac{1}{(m_{G_{239}}/m_{C_{239}}+1+m_{S_{239}}/m_{C_{239}})}+R_{S_{240}}\frac{1}{(m_{G_{239}}/m_{S_{239}}+m_{C_{239}}/m_{S_{239}}+1)}$$

$$=R_{G_{240}}\frac{m_{G_{239}}}{\left(m_{G_{239}}+m_{C_{239}}+m_{S_{239}}\right)}+R_{C_{240}}\frac{m_{C_{239}}}{(m_{G_{239}}+m_{C_{239}}+m_{S_{239}})}+R_{S_{240}}\frac{m_{S_{239}}}{(m_{G_{239}}+m_{C_{239}}+m_{S_{239}})}$$

where

$$R_{G_{240}}=\frac{m_{G_{240}}}{m_{G_{239}}}, R_{C_{240}}=\frac{m_{C_{240}}}{m_{C_{239}}}, R_{S_{240}}=\frac{m_{S_{240}}}{m_{S_{239}}}$$

and

$$F_{G_{239}}=\frac{m_{G_{239}}}{\left(m_{G_{239}}+m_{C_{239}}+m_{S_{239}}\right)},$$

$$F_{C_{239}}=\frac{m_{C_{239}}}{(m_{G_{239}}+m_{C_{239}}+m_{S_{239}})}, F_{S_{239}}=\frac{m_{S_{239}}}{(m_{G_{239}}+m_{C_{239}}+m_{S_{239}})}$$

and

$$F_{G_{239}}+F_{C_{239}}+F_{S_{239}}=1$$

$$R_{240/239}=R_{G_{240}}F_{G_{239}}+R_{C_{240}}F_{C_{239}}+R_{S_{240}}F_{S_{239}}$$

And similarly, for ^244^Pu:

$$R_{244/239}=R_{G_{244}}F_{G_{239}}+R_{C_{244}}F_{C_{239}}+R_{S_{244}}F_{S_{239}}$$

Substituting for,

$$F_{G_{239}}=1-F_{C_{239}}-F_{S_{239}}$$

$$R_{240/239}=R_{G_{240}}\left(1-F_{C_{239}}-F_{S_{239}}\right)+R_{C_{240}}F_{C_{239}}+R_{S_{240}}F_{S_{239}}$$

And,

$$R_{244/239}=R_{G_{244}}\left(1-F_{C_{239}}-F_{S_{239}}\right)+R_{C_{244}}F_{C_{239}}+R_{S_{244}}F_{S_{239}}$$

Simplifying,

$$R_{240/239}=R_{G_{240}}-R_{G_{240}}F_{C_{239}}-R_{G_{240}}F_{S_{239}}+R_{C_{240}}F_{C_{239}}+R_{S_{240}}F_{S_{239}}$$

$$R_{244/239}=R_{G_{244}}-R_{G_{244}}F_{C_{239}}-R_{G_{244}}F_{S_{239}}+R_{C_{244}}F_{C_{239}}+R_{S_{244}}F_{S_{239}}$$

Hence

$$R_{240/239}=R_{G_{240}}+F_{C_{239}}\left(R_{C_{240}}-R_{G_{240}}\right)+F_{S_{239}}\left(R_{S_{240}}-R_{G_{240}}\right)$$

$$R_{244/239}=R_{G_{244}}+F_{C_{239}}\left(R_{C_{244}}-R_{G_{244}}\right)+F_{S_{239}}\left(R_{S_{244}}-R_{G_{244}}\right)$$

Two simultaneous equations follow,

$$R_{\frac{240}{239}}/\left(R_{S_{240}}-R_{G_{240}}\right)=R_{G_{240}}/\left(R_{S_{240}}-R_{G_{240}}\right)+F_{C_{239}}\left(R_{C_{240}}-R_{G_{240}}\right)/\left(R_{S_{240}}-R_{G_{240}}\right)+F_{S_{239}}$$

$$R_{\frac{244}{239}}/\left(R_{S_{244}}-R_{G_{244}}\right)=R_{G_{244}}/\left(R_{S_{244}}-R_{G_{244}}\right)+F_{C_{239}}\left(R_{C_{244}}-R_{G_{244}}\right)/\left(R_{S_{244}}-R_{G_{244}}\right)+F_{S_{239}}$$

Eliminating $F_{S_{239}}$ gives,

$$\frac{R_{\frac{240}{239}}}{R_{S_{240}}-R_{G_{240}}}-\frac{R_{\frac{244}{239}}}{R_{S_{244}}-R_{G_{244}}}=\frac{R_{G_{240}}}{R_{S_{240}}-R_{G_{240}}}+\frac{F_{C_{239}}\left(R_{C_{240}}-R_{G_{240}}\right)}{R_{S_{240}}-R_{G_{240}}}-\frac{R_{G_{244}}}{R_{S_{244}}-R_{G_{244}}}-\frac{F_{C_{239}}\left(R_{C_{244}}-R_{G_{244}}\right)}{R_{S_{244}}-R_{G_{244}}}$$

$$\frac{R_{\frac{240}{239}}-R_{G_{240}}}{R_{S_{240}}-R_{G_{240}}}+\frac{R_{G_{244}}-R_{\frac{244}{239}}}{R_{S_{244}}-R_{G_{244}}}=F_{C_{239}}\left[\frac{\left(R_{C_{240}}-R_{G_{240}}\right)}{R_{S_{240}}-R_{G_{240}}}-\frac{\left(R_{C_{244}}-R_{G_{244}}\right)}{R_{S_{244}}-R_{G_{244}}}\right]$$

Such that the fraction of ^239^Pu associated with the ^240^Pu/^239^Pu fraction consistent with Chernobyl, $F_{C_{239}}$, is,

$F_{C_{239}}=\left[\frac{R_{\frac{240}{239}}-R_{G_{240}}}{R_{S_{240}}-R_{G_{240}}}+\frac{R_{G_{244}}-R_{\frac{244}{239}}}{R_{S_{244}}-R_{G_{244}}}\right]/\left[\frac{\left(R_{C_{240}}-R_{G_{240}}\right)}{R_{S_{240}}-R_{G_{240}}}-\frac{\left(R_{C_{244}}-R_{G_{244}}\right)}{R_{S_{244}}-R_{G_{244}}}\right]$ (S2)
